# Pre-depletion of TRBC1+ T cells promotes the therapeutic efficacy of anti-TRBC1 CAR-T for T-cell malignancies

**DOI:** 10.1186/s12943-020-01282-7

**Published:** 2020-11-21

**Authors:** Chaoting Zhang, Heyilimu Palashati, Zhuona Rong, Ningjing Lin, Luyan Shen, Ying Liu, Shance Li, Bentong Yu, Wenjun Yang, Zheming Lu

**Affiliations:** 1 Key Laboratory of Carcinogenesis and Translational Research (Ministry of Education/Beijing), Laboratory of Biochemistry and Molecular Biology, Peking University Cancer Hospital & Institute, Beijing, 100142 China; 2 Key Laboratory of Carcinogenesis and Translational Research, Departments of Lymphoma, Radiology and Nuclear Medicine, Peking University Cancer Hospital and Institute, Beijing, 100142 China; 3 Key Laboratory of Carcinogenesis and Translational Research (Ministry of Education/Beijing), Laboratory of Genetics, Peking University Cancer Hospital & Institute, Beijing, 100142 China; 4 Department of Thoracic Surgery, The First Affiliated Hospital of Nanchang University, Jiangxi, 330006 China; 5 Key Laboratory of Fertility Preservation and Maintenance, School of Basic Medicine and the General Hospital, Ningxia Medical University, Yinchuan, 750004 Ningxia China

**Keywords:** T cell receptor β-chain constant region 1, CAR-T, T-cell malignancy

## Abstract

Targeting T cell receptor β-chain constant region 1 (TRBC1) CAR-T could specifically kill TRBC1+ T-cell malignancies. However, over-expressed CARs on anti-TRBC1 CAR transduced TRBC1+ T cells (CAR-C1) bound to autologous TRBC1, masking TRBC1 from identification by other anti-TRBC1 CAR-T, and moreover only the remaining unoccupied CARs recognized TRBC1+ cells, considerably reducing therapeutic potency of CAR-C1. In addition, co-culture of anti-TRBC1 CAR-T and TRBC1+ cells could promote exhaustion and terminal differentiation of CAR-T. These findings provide a rationale for pre-depleting TRBC1+ T cells before anti-TRBC1 CAR-T manufacturing.

**Supplementary Information:**

The online version contains supplementary material available at 10.1186/s12943-020-01282-7.

## Background

Chimeric antigen receptor (CAR) T cells showed remarkable efficacy for the treatment of B-cell malignancies and have been approved by the US Food and Drug Administration for the treatment of relapsed/refractory B-cell acute lymphoblastic leukemia (B-ALL) and diffuse large B-cell lymphoma (DLBCL) [1, 2]. However, the development of CAR-T cells against T-cell malignancies seems more challenging due to the similarities between the normal, malignant and therapeutic T cells, which could result into CAR-T cell fratricide, T cell aplasia, and contamination of CAR-T cell products with malignant T cells [3, 4].

**Fig. 1:**
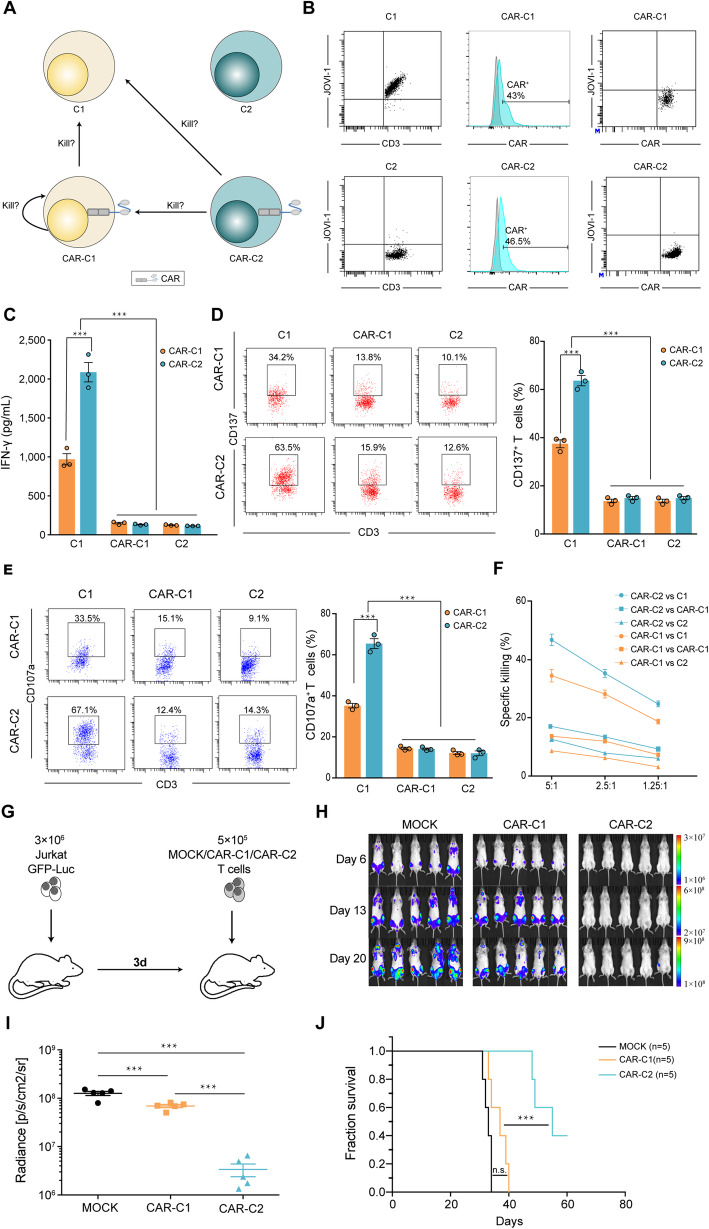
Effector functions of TRBC1^+^ and TRBC2^+^ cells genetically engineered with anti-TRBC1 CAR. **a** The categories and relationship of T cells following transduction with anti-TRBC1 CAR. TRBC1^+^ cells, C1; TRBC2^+^ cells, C2; anti-TRBC1 CAR transduced TRBC1^+^ cells, CAR-C1; anti-TRBC1 CAR transduced TRBC2^+^ cells, CAR-C2. **b** TRBC1 expression and CAR transduction efficacy of TRBC1-sorted and TRBC1-depleted T cells as well as CAR and TRBC1 expression of CAR-C1 and CAR-C2 analyzed by flow cytometry. **c** IFN-γ secretion by CAR-C1 and CAR-C2 against C1, CAR-C1 or C2 after 24-h co-culture. **d-e** Left, representive FACS profile of CD137 and C107a expression on CAR-C1 and CAR-C2 co-cultured with C1, CAR-C1 or C2. Right, percentages of CD137- and C107a-positive CAR-C1 and CAR-C2 following co-culture with C1, CAR-C1 or C2. **f** Cytotoxic activities of CAR-C1 and CAR-C2 against C1, CAR-C1 or C2 were examined by standard CFSE-based cytotoxity assays at several effector/target (E/T) ratios. **g** Scheme of the xenograft model. NOG mice (*n* = 5/group) were IV injected with 3 × 10^6^ Luc/GFP–expressing Jurkat cells followed 3 days after by a single IV injection of 5 × 10^5^ MOCK, CAR-C1 or CAR-C2. **h** IVIS imaging of tumor burden monitored by BLI at the indicated time points following MOCK, CAR-C1 or CAR-C2 T cell injection (day 0). **i** Radiance of individual mice at day 20 following MOCK, CAR-C1 or CAR-C2 T cell injection. *n* = 5 mice per group. **j** Kaplan-Meier survival curve of mice injected with mock, CAR-C1 or CAR-C2 T cells. ***P < 0.001 and n.s., not significant

An innovative treatment option for T-cell malignancy was proposed that targeting T cell receptor β-chain constant region 1 (TRBC1) CAR-T could specifically identify and kill TRBC1^+^ T-cell malignancies, since either TRBC1 or TRBC2 is mutually exclusively expressed in T cells and moreover proportion of TRBC1^+^ T cells varies between 25 and 47% in healthy individuals, but malignant T cells are clonally TRBC1 positive or negative [5, 6]. Thus, anti-TRBC1 CAR-T cells could specifically kill TRBC1^+^ malignant T cells while sparing TRBC2^+^ normal T cells. However, anti-TRBC1 CAR gene could probably be inadvertently transferred into TRBC1^+^ malignant T cells during CAR-T cell manufacturing, and its product could in cis bind to autologous TRBC1 on the surface of malignant T cells, which could result into masking TRBC1 from identification by and mediating resistance to anti-TRBC1 CAR-T and meanwhile weaken effector function of anti-TRBC1 CAR transduced TRBC1^+^ cells. Following transduction of T cells with lentivirus encoding anti-TRBC1 CAR, all T cells could be categorized into TRBC1^+^ cells (C1), TRBC2^+^ cells (C2), anti-TRBC1 CAR transduced C1 cells (CAR-C1) and anti-TRBC1 CAR transduced C2 cells (CAR-C2) (Fig. 1a). Thus, it is interesting to evaluate whether both C1 and CAR-C1 could be identified and killed by CAR-C1 and CAR-C2 (Fig. 1a).

## Results and discussions

To evaluate whether C1 and CAR-C1 could be identified and killed by CAR-C1 and CAR-C2, we first sorted donor T cells into TRBC1^+^ and TRBC1^−^ (designated as C2) fractions using magnetic beads. A portion of C1 or C2 were used as target cells and other C1 and C2 from the same donor were genetically engineered with anti-TRBC1 CAR to obtain CAR-C1 and CAR-C2 as effect cells. We confirmed that transduction efficacy of anti-TRBC1 CAR was similar on C1 and C2, and moreover TRBC1 was not detected on CAR-C1 through flow cytometry (Fig. 1b). Since primed T cells could increase CD137 expression and IFN-γ secretion, and moreover cytotoxic T cells could express CD107 and mediated killing of target cells, these markers could be used to detect activation and cytolytic activity of T cells. We found that CAR-C2 than CAR-C1 showed higher level of IFN-γ production and CD137 expression when co-cultured with C1 but not CAR-C1 or C2 (Fig. 1c and d). In flow cytometry–based cytotoxicity assays, CAR-C2 and CAR-C1 both specifically killed C1 but not CAR-C1 or C2, more so in CAR-C2 than CAR-C1 (Fig. 1e and f).

We next evaluated the anti-tumour activity of CAR-C1 and CAR-C2 in vivo using Luc-expressing Jurkat T-ALL cells. NOG mice were transplanted with 3 × 10^6^ Luc-expressing Jurkat cells 3 days before IV infusion of 5 × 10^5^ CAR-C1, CAR-C2 or MOCK T cells (Fig. 1g). Consistent with the in vitro observation, CAR-C1 induced transient tumour regression, but tumours re-progressed rapidly. In contrast, mice treated with an equal number of CAR-C2 exhibited significantly higher ani-tumour ability with significantly prolonged survival (*P* < 0.001) (Fig. 1h-j).

**Fig. 2:**
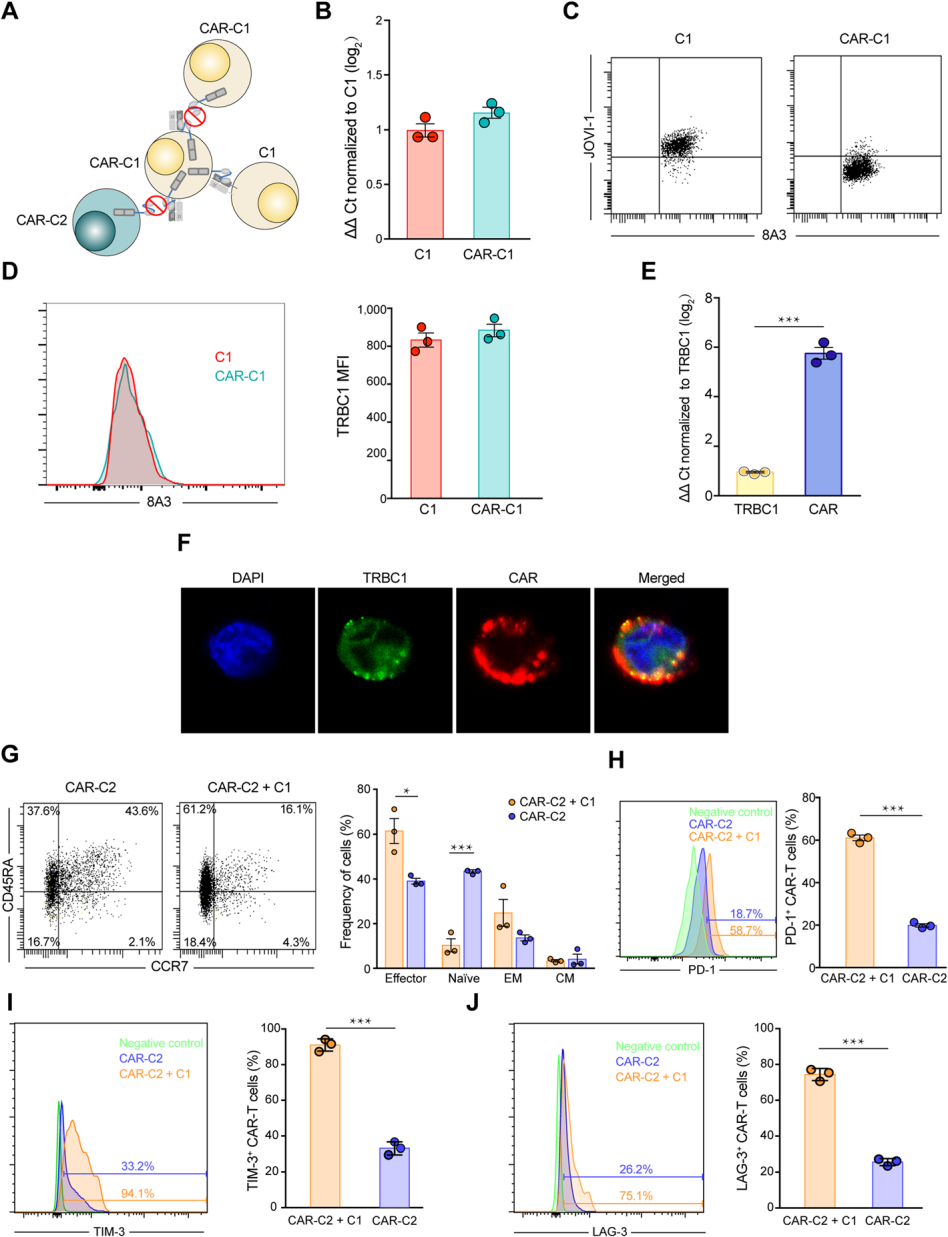
The cause for undetected TRBC1 and lesser effector function of CAR-C1. **a** Due to higher expression level of CAR than TRBC1 on CAR-C1, some CARs in cis bind to autologous TRBC1 on CAR-C1, resulting into masking TRBC1 from identification by other anti-TRBC1 CAR-T and meanwhile occupying these CARs, and thus only the remaining unoccupied CARs target TRBC1. **b** TRBC1 mRNA expression is maintained in CAR-C1 as compared to C1, as determined by qRT-PCR (ΔΔ Ct normalized to C1). **c** TRBC1 on C1 is detectable using both mAb 8A3 targeting TCRβ-chain constant region and mAb JOVI-1 from which the anti-TRBC1 CAR was derived, but TRBC1 on CAR-C1 cells is only recognized by mAb 8A3. **d** Left, representive FACS profile of TRBC1 expression on CAR-C1 and C1. Right, MFI of TRBC1 on CAR-C1 and C1. **e** Expression level of CAR was significantly higher than TRBC1 on CAR-C1 determined by qRT-PCR analysis (ΔΔ Ct normalized to TRBC1). **f** Confocal imaging of CAR-C1 using FITC-conjugated anti-TRBC1 antibody (green), TRITIK-conjugated anti-FLAG antibody (red), and DAPI (blue). Scale bars, 5 μm. **g** Left, representive FACS profile of CD45RA and CCR7 expression on CAR-C2 after 6-day culture alone or co-culture with C1. Right, percentages of naïve (CD45RA^+^ CCR7^+^), effector (CD45RA^+^ CCR7^−^), effector memory (CD45RA^−^ CCR7^−^) and central memory (CD45RA^−^ CCR7^+^) CAR-C2 cells. **h-j** Left, representive FACS profile of PD-1 (**h**), TIM-3 (**i**) and LAG-3 (**j**) expression on CAR-C2 after 6-day culture alone or co-culture with C1. Right, percentage of PD-1 (**h**), TIM-3 (**i**) and LAG-3 (**j**) positive CAR-C2. *P < 0.05, ***P < 0.001. Data are representative of three independent experiments

To investigate why CAR-C1 than CAR-C2 demonstrated lesser killing ability against C1 and moreover neither of them could identify and kill CAR-C1, we hypothesize that since expression abundance of anti-TRBC1 CAR is significantly higher than TRBC1 on CAR-C1, a proportion of CARs in cis bind to autologous TRBC1 on CAR-C1, masking TRBC1 from identification by other anti-TRBC1 CAR-T, and meanwhile only the remaining unoccupied CARs identify C1, weakening effector function of CAR-C1 (Fig. 2a).

We first found that TRBC1 mRNA expression was preserved in CAR-C1 as compared to C1 determined by qRT-PCR analysis (Fig. 2b). We further confirmed via flow cytometry that TRBC1 on CAR-C1 was detectable by anti-TRBC monoclonal antibody (mAb) 8A3 targeting not the same epitope recognized by mAb JOVI-1 from which the anti-TRBC1 CAR was derived (Fig. 2c), and moreover expression level of TRBC1 protein was similar on CAR-C1 and C1 (Fig. 2d). Meanwhile, qRT-PCR analysis demonstrated that expression level of CAR was significantly higher than TRBC1 in CAR-C1 and moreover confocal microscopy further confirmed that colocalization of anti-TRBC1 CAR and TRBC1 on the cell surface of CAR-C1 (Fig. 2e and f). These findings supported that TRBC1 molecules were still expressed on the surface of CAR-C1 but in cis bound by a proportion of anti-TRBC1 CARs, masking TRBC1 from identification by other anti-TRBC1 CAR-T, and meanwhile only the remaining unoccupied CARs identified C1, weakening effector function of CAR-C1.

In addition, contaminating TRBC1^+^ malignant cells during anti-TRBC1 CAR-T manufacturing not only produced CAR-C1 which was resistant to anti-TRBC1 CAR-T and had lesser killing ability, but were expected to accelerate exhaustion and terminal differentiation of anti-TRBC1 CAR-T with limited in vivo persistence due to continuous (tonic) ligand-driven CAR stimulation [7, 8]. Co-culture of CAR-C2 with C1 in a 2:1 ratio (physiological condition) for 6 days revealed lower and higher percent of naïve and effect CAR-C2 cells, respectively, compared to solo culture of CAR-C2 (Fig. 2g). In addition, the co-culture of CAR-C2 and C1 exhibited increasing expression of PD-1, TIM-3 and LAG-3 in CAR-C2 (Fig. 2h-j). These findings suggested that compared with unfractionated T cells, TRBC1-depleted T cells genetically engineered with anti-TRBC1 CAR not only avoided resistance to anti-TRBC1 CAR-T, but reduced exhaustion and terminal differentiation.

## Conclusions

Although anti-TRBC1 CAR-T appeared a promising approach for T-cell malignancy, unfractioned T cells transduced to express anti-TRBC1 CAR could not only produce CAR-C1 cells which had lesser killing ability against TRBC1+ malignant T cells and moreover were resistant to anti-TRBC1 CAR-T, but contaminate TRBC1+ cells which promoted exhaustion and terminal differentiation of anti-TRBC1 CAR-T. Therefore, it was necessary to pre-deplete TRBC1+ T cells, even if allogeneic T cells were used for anti-TRBC1 CAR-T manufacturing for patients without sufficient autologous T cells.

## Supplementary Information

**Additional file 1.**

## Data Availability

The datasets used and analysed during the current study are available from the corresponding author on reasonable request.

## Abbreviations

CAR: Chimeric antigen receptor
B-ALL: B-cell acute lymphoblastic leukemia
DLBCL: diffuse large B-cell lymphoma
TRBC1: T cell receptor β-chain constant region 1
C1: TRBC1^+^ cells
C2: TRBC2^+^ cells
CAR-C1: anti-TRBC1 CAR transduced C1 cells
CAR-C2: anti-TRBC1 CAR transduced C2 cells
