# Supplementary material for: Pre-depletion of TRBC1+ T cells promotes the therapeutic efficacy of anti-TRBC1 CAR-T for T-cell malignancies
Source: Mol Cancer. 2020 Nov 21;19:162. doi: 10.1186/s12943-020-01282-7 (PMC7679992; doi:10.1186/s12943-020-01282-7)
Supplement: Supplementary file 1 — Additional file 1. [file 12943_2020_1282_MOESM1_ESM.docx]

**Methods**

**CAR design and transduction**

The anti-TRBC1 single chain variable fragment (scFv) sequence based on the JOVI-1 epitope [[1](#_ENREF_1)] was generated by using commercial gene synthesis (GenScript) and cloned into the backbone of a second-generation CAR with CD8 hinge, CD8 transmembrane, 4-1BB costimulatory, and CD3ζ signaling domains as well as flag tag which is used to detect anti-TRBC1 CAR expression [[2](#_ENREF_2), [3](#_ENREF_3)]. T cells were activated by OKT3 (50ng/mL, ACRO, USA) and anti-CD28 antibodies（1ug/mL, Bio-Tool, China）, transduced with lentiviral vectors and expanded in the X-VIVO 15 (Lonza, USA) with the presence of IL2 (50U/mL, Perprotech, USA), IL7 (5ng/mL, Perprotech, USA) and IL15(5ng/mL, Perprotech, USA).

**Flow cytometry**

Flow cytometry was performed by BD FACS AriaⅡ (BD Biosciences). The following anti-human antibodies (BD Biosciences; clone IDs are given in parentheses) were used: CD3 (UCHT1), CD4 (RPA-P4), CD8 (SK1), CCR7 (150503), CD45RA (HI100), TCR βF1 (8A3), Cβ1 TCR (JOVI.1), CD107a (H4A3), CD137 (4B4-1), PD-1 (EH12.1), TIM-3 (7D3), and LAG-3 (T48-530). Monoclonal anti-FLAG (M2, Sigma-Aldrich) was used to detect anti-TRBC1 CAR expression [[2](#_ENREF_2)]. Fixable Viability Stain 780 (FVS780, BD Biosciences) was used to exclude the dead cells.

**Identification of T cell** **differentiation subsets**

T cells subsets were defined as naïve (CD45RA^+^ CCR7^+^), effector (CD45RA^+^ CCR7^-^), effector memory (CD45RA^-^ CCR7^-^) and central memory (CD45RA^-^ CCR7^+^).

**Cell sorting with magnetic bead selection**

Peripheral blood mononuclear cells from healthy donors were isolated by Ficoll-Paque gradient centrifugation and then T cells were selected by negative bead selection using Dynabeads® Untouched™ Human T Cells (Life Technologies) according to the manufacturer’s instructions. TRBC1^+^ and TRBC1^-^ T cells were separated by magnetic isolation using anti-TCR Cb1/Biotin (JOVI-1, Ansell) and CELLection™ Biotin Binder Kit (Thermo Fisher Scientific) according to the manufacturer’s instructions. Anti-TRBC1 CAR-positive T cells were separated by magnetic isolation using anti-flag/Biotin (M2, Sigma-Aldrich) and CELLection™ Biotin Binder Kit (Thermo Fisher Scientific) according to the manufacturer’s instructions. Selection and depletion was performed twice to increase purity.

**Cytokine production quantification**

Target cells were seeded at 1 ×10^5^ cells/well in a 96-well plate and effector cells were co-incubated at E:T ratio of 1:1 for 24 hours at 37˚C. Human IFN-gamma DuoSet ELISA (R&D Systems) was used to measure the cytokine concentrations in the culture supernatant.

**FACS-based cytotoxicity assays**

Target cells were prelabled with carboxyfluorescein succinimidyl ester (CFSE, BD Biosciences) according to the manufacturer’s instructions and then co-incubated with effector cells at 37˚C at the E: T ratio of 1.25:1, 2.5:1, 5:1 for 6 hours. After the co-incubation, 1μg/mL propidium iodide (PI, BD Biosciences) was added and cells were stained for 20 min at 4 ˚C. And then BD Accuri C6 (BD Biosciences) was used to analyze the samples.

**Quantitative real-time PCR**

Total RNA was extracted from cells using an RNAeasy isolation kit (Qiagen) and was converted to cDNA with GoScript™ Reverse Transcription System (Promega). Quantitative PCR was performed on Step One Real-Time PCR System (Applied Biosystems) with SYBR Green Real time PCR Master Mix(TOYOBO) in triplicate wells according to the manufacturer’s instructions. Following primer sets are used: β-actin: forward 5^’^-AGAGCTACGAGCTGCCTGAC-3’,reverse 5’-TAGTTTCGTGGATGCCACAGG-3’;TRBC1: forward 5’- CGCTGTGTTTGAGCCATCAG-3’,reverse5’- GCTGACCAGCACAGCATACA-3’; anti-TRBC1 CAR: forward5’- GTGCACAGCAACGGCAATAC-3’,reverse 5’- GCACGCCTGGGAATCTGTTA-3’.TRBC1 and anti-TRBC1 CAR expression was normalized to β-actin expression levels.

**Confocal microscopy**

CAR-C1 cells were stained with rabbit anti–FLAG (ab1162, Abcam) for 45 min followed by secondary antibody TRITC-conjugated goat anti–rabbit IgG (ORIGENE) for 40 min at room temperature. And CAR-C1 cells were fixed and permeabilized with Fixation/Permeabilization Solution Kit (BD) and then were stained with mouse anti-TCR beta constant region (ab171088, Abcam) for 1 h and secondary antibody FITC-conjugated goat anti–mouse IgG (ORIGENE) for 40 min at room temperature. Finally, the cells were counterstained with DAPI and used for confocal images, using a 63 × oil immersion lens on a Leica SP2 confocal microscope (Leica Microsystems) at Core Laboratory, Peking University Cancer Hospital & Institute.

**Mouse xenograft model of T cell malignancy**

6- to 8-week old male NOG mice (Beijing Vital River Laboratory Animal Technology Co., Ltd.) were engrafted with 3×10^6^ Luc/GFP–expressing Jurkat cells (Jurkat-GFP-Luc) by IV injection, and 5×10^5^ of mock or TRBC1 CAR-T cells were injected IV 3 days later. To measure luminescence, mice were injected with 150 mg/kg of D-luciferin intraperitoneally, and tumor burden was followed by measuring luminesce in an IVIS Imaging system (Caliper LifeSciences). Living Image software (PerkinElmer) was used to visualize and calculate total luminescence.

**Statistics**

Unless otherwise noted, data are summarized as mean ± s.e.m. The unpaired Student’s t-test and one-way ANOVA with Dunnett’s test for multiple comparisons were used to determine statistically significant differences when two or three groups were compared. Survival curves were generated using the Kaplan–Meier method and compared by Cox proportional hazard model with bootstrap variance. Statistical analysis was performed on using STATA version 12.0 (STATA Corporation, College Station, TX, USA). Differences with a *P* value < 0.05 were considered statistically significant. Asterisks are used in each figure to represent *P* values (**P* < 0.05, ****P* < 0.001).

1. Viney JL, Prosser HM, Hewitt CR, Lamb JR, Owen MJ: **Generation of monoclonal antibodies against a human T cell receptor beta chain expressed in transgenic mice**. *Hybridoma* 1992, **11**(6):701-713.

2. Berahovich R, Xu S, Zhou H, Harto H, Xu Q, Garcia A, Liu F, Golubovskaya VM, Wu L: **FLAG-tagged CD19-specific CAR-T cells eliminate CD19-bearing solid tumor cells in vitro and in vivo**. *Front Biosci (Landmark Ed)* 2017, **22**:1644-1654.

3. Imai C, Mihara K, Andreansky M, Nicholson I, Pui C, Geiger T, Campana D: **Chimeric receptors with 4-1BB signaling capacity provoke potent cytotoxicity against acute lymphoblastic leukemia**. *Leukemia* 2004, **18**(4):676-684.
